# Combinations of Plant Essential Oil Based Terpene Compounds as Larvicidal and Adulticidal Agent against *Aedes aegypti* (Diptera: Culicidae)

**DOI:** 10.1038/s41598-019-45908-3

**Published:** 2019-07-01

**Authors:** Riju Sarma, Kamal Adhikari, Sudarshana Mahanta, Bulbuli Khanikor

**Affiliations:** 1 Research Scholar, Department of Zoology, Gauhati University, Guwahati, Assam India; 2 Assistant Professor, Department of Zoology, Gauhati University, Guwahati, Assam India

**Keywords:** Entomology, Secondary metabolism

## Abstract

Insecticidal plant-based compound(s)in combinations may show synergistic or antagonistic interactions against insect pest. Considering the rapid spread of the *Aedes* borne diseases and increasing resistance among *Aedes* population against conventional insecticides, twenty-eight combinations of plant essential oil-based terpene compounds were prepared and tested against larval and adult stages of*Aedes aegypti*. Initially five plant essential oils (EOs) were assessed for their larvicidal and adulticidal efficacy and two of their major compounds from each EO were identified from GC-MS results. Identified major compounds namely Diallyldisulfide, Diallyltrisulfide, Carvone, Limonene, Eugenol, Methyl Eugenol, Eucalyptol, Eudesmol and α-pinene were purchased and tested individually against *A*. *aegypti*. Binary combinations of these compounds were then prepared using sub-lethal doses, tested and their synergistic and antagonistic effects were determined. The best larvicidal compositions were obtained while Limonene was mixed with Diallyldisulfide and the best adulticidal composition was obtained while Carvone was mixed with Limonene. Commercially used synthetic larvicide “Temephos” and adulticide “Malathion” were tested individually and in binary combinations with the terpene compounds. The results revealed that the combination of Temephos and Diallyldisulfide and combination of Malathion and Eudesmol were the most effective combination. These effective combinations bear potential prospect to be used against *Aedes aegypti*.

## Introduction

Plant essential oils (EO) are secondary metabolites comprising different bioactive compounds and have been getting importance as alternative to synthetic insecticide. They are not only ecofriendly and user-friendly but being a mixture of different bioactive compounds, also offer less chance of resistance development^1^. With the accessibility of GC-MS technique, researchers explored the constituent compounds of different plant EOs and more than 3000 compounds from 17500 aromatic plants have been identified^2^ most of which were tested for their insecticidal properties and reported to have insecticidal effects^3,4^. Some of the studies highlighted equal or higher toxicity of major constituent compound than its crude EO. But application of a single compound may again leave chance for resistance development like that of chemical insecticides^5,6^. Hence, emphasisis are now-a- days given to prepare mixtures of EO based compounds to enhance insecticidal effects as well as to reduce the probability of development of resistance by the targeted pest population. The individual active compound present in an EO may exhibit synergistic or antagonistic effects in combinations representing the overall activity of the EO and the fact is highlighted well in studies carried out by previous workers^7,8^. In vector control programme also, EOs and their constituents are incorporated. Mosquitocidal activities of EOs were extensively studied upon *Culex* and *Anopheles*. Few studies also attempted to formulate effective insecticide by combining different botanicals with commercially used synthetic insecticide aiming to increase the overall toxicity as well as to minimize the side effects^9^. But study of such formulated compounds against *Aedes aegypti* is still scanty. Advancement of medical science with development of medication and vaccination help to handle some of the vector transmitted diseases. But presence of different serotypes of viruses transmitted by *Aedes aegypti* makes vaccination programme unsuccessful. Thus, in cases of such diseases, vector control programme is the only option to prevent disease transmission. In the present context, control of *Aedes aegypti* is very much important as it is the key transmitter of different viruses and their serotypes causing dengue, zika, dengue hemorrhagic fever, yellow fever etc. Most notably the number of cases of almost all of these *Aedes aegypti* borne diseases has been increasing globally every year and the trend is increasing. Therefore, it is an urgent need in this situation to develop ecofriendly and effective measures to control *Aedes aegypti* population. In this respect EOs,constituent compounds and their combinations are potential candidate. Therefore, the present study was attempted to find out effective synergistic combinations of major plant EO compounds of five plants having insecticidal property namely *Mentha piperita*, *Ocimum sanctum*, *Eucalyptus maculata*, *Allium sativum* and *Callistemon linearis* against *Aedes aegypti*.

## Results

### Larvicidal activity of the EO

All the selected EO showed potential larvicidal activity with LC50 for 24 h lies between 0.42 to 163.65 ppm against *Aedes aegypti*. The highest larvicidal activity was recorded for the EO of *Mentha piperita* (Mp)having LC50 value of 0.42 ppm at 24 h followed by *Allium sativum*(As) having LC50 value of 16.19 ppm at 24 h (Table 1).

**Table 1:** LC50 of the selected EO against 4^th^ instar larvae and adults of *Aedes aegypti*.

Sl No	EO	Bioassay	Time (hour)	LC50 value (ppm)	Regression equation	95%confidence level		χ^2^ value
						Lower level	Upper level	
1	Os	Larvicidal	24	27.25	Y = 0.93 + 2.83x	2.202	3.654	12.732
			48	23.48	Y = 1.02 + 2.90x	2.481	4.200	9.782
			72	22.88	Y = 1.08 + 2.88x	2.487	4.210	8.900
		Adulticidal	24	—	—	—	—	—
			48	—	—	—	—	—
			72	—	—	—	—	—
2	Em	Larvicidal	24	49.09	Y = 0.27 + 2.80x	2.069	3.368	8.475
			48	37.64	Y = 0.46 + 2.89x	2.164	3.573	6.152
			72	34.49	Y = 0.79 + 2.74x	2.032	3.267	14.529
		Adulticidal	24	101.91	Y = −2.64 + 3.81	2.477	4.366	29.689
			48	26.76	Y = 2.18 + 1.98x	1.435	2.534	22.032
			72	21.96	Y = 2.67 + 1,74x	1.254	2.253	27.932
3	As	Larvicidal	24	16.19	Y = 1.56 + 2.84x	3.552	6.978	7.649
			48	7.57	Y = 3.01 + 2.23x	1.681	3.079	8.040
			72	7.57	Y = 3.01 + 2.23x	1.681	3.079	8.040
		Adulticidal	24	120.16	Y = 2.67 + 1.11x	0.851	1.393	12.914
			48	66.35	Y = 3.30 + 0.94x	0.695	1.182	66.35
			72	29.68	Y = 3.71 + 0.88x	0.647	1.108	25.623
4	Mp	Larvicidal	24	0.42	Y = 5.8 + 2.12x	2.289	3.256	12.654
			48	0.16	Y = 6.43 + 1.80x	2.073	3.097	16.301
			72	0.08	Y = 6.59 + 1.44x	1.184	2.032	23.046
		Adulticidal	24	118	Y = 0.48 + 2.19x	1.666	2.724	18.893
			48	51.57	Y = 1.80 + 1.87x	1.427	2.315	23.788
			72	42.35	Y = 0.67 + 2.66x	1.877	3.278	14.033
5	Cl	Larvicidal	24	163.65	Y = 1.05 + 1.79x	1.341	2.221	21.179
			48	80.53	Y = 2.51 + 1.30x	0.972	1.629	47.519
			72	76.66	Y = 2.32 + 1.42x	1.078	1.768	53.529
		Adulticidal	24	23.37	Y = 1.54 + 2.53x	1.649	3.029	10.016
			48	18.47	Y = 2.37 + 2.08x	1.337	2.561	6.572
			72	5.02	Y = 3.71 + 1.84x	0.821	2.792	10.855

### Adulticidal activity of the EO

Except for the EO of *Ocimum sanctum* (Os), the rest four selected EO showed clear adulticidal effects with LC50 value lies between 23.37 ppm to 120.16 ppm at 24 h exposure period. The highest adulticidal efficacy was recorded for the EO of *Callistemon linearis* (Cl) with LC50 value of 23.37 ppm at 24 h post exposure period followed by *Eucalyptus maculata* (Em) with LC50 value of 101.91 ppm (Table 1). On the other hand, the LC50 value for the Os was not determined as maximum 53% percent mortality was recorded at the highest dose applied (Supplementary Fig. 3).

### Analysis of effective EO components

**Figure 1:**
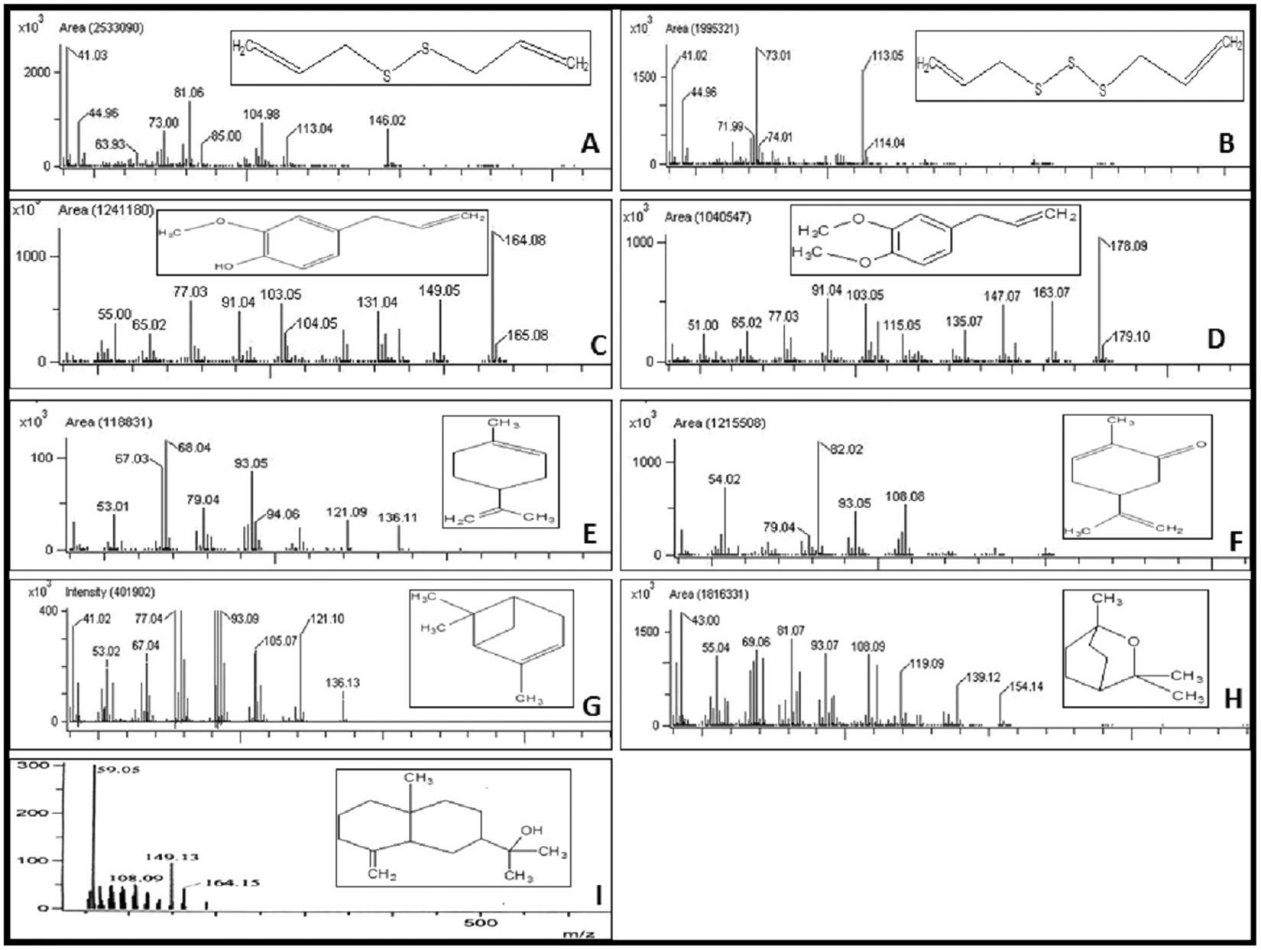
Mass- spectrometric result of major terpene compounds of the selected essential oils (**A**- Diallyldisulfide; **B**- Diallyltrisulfide; **C**- Eugenol; **D**- Methyl Eugenol; **E**- Limonene; **F**- Carvone; **G**- α- pinene; **H**- Eucalyptol; **I**- Eudesmol).

Based on NIST library database results, area percentage of GC- chromatogram and MS spectral results,two major constituent compounds from each EO were identified and selected (Table 2). For EO of As, the major compounds identified were Diallyldisulfide and Diallyltrisulfide, for the EO of Mp, the compounds were Carvone and Limonene and for the EO of Em, the compounds were Eudesmol and Eucalyptol. For the EO of Os the major compounds identified were Eugenol and MethylEugenol and for the EO of Cl the compounds identified were Eucalyptol and α- pinene (Fig. 1, Supplementary Figs 5–8, Supplementary Tables 1–5).

**Table 2:** Two major constituent compounds of the selected plant Eos.

Sl no	EO	Major compound	Chemical structure	Chemical formula	Molecular weight	Area (%)	Retention time (min)
1	As	Diallyldisulfide		C_6_H_10_S_2_	146	8.51	4.95
		Diallyltrisulfide		C_6_H_10_S_3_	178	7.75	8.58
2	Mp	Carvone		C_10_H_14_0	150	79.6	7.96
		Limonene		C_10_H_16_	136	6.65	4.43
3	Os	Eugenol		C_10_H_12_O_2_	164	52.30	11.86
		Methyl Eugenol		C_11_H_14_O_2_	178	14.87	12.48
4	Em	Eudesmol		C_15_H_26_O	222.37	31.80	14
		Eucalyptol		C_10_H_18_O	154	17.64	5.6
5	Cl	α- pinene		C_10_H_16_	136	9.28	4.43
		Eucalyptol		C_10_H_18_O	154	32.6	6.24

### Bioassays of individual major terpene compounds against *A*. *aegypti*

#### Larvicidal activity

Total nine compounds (Diallyldisulfide, Diallyltrisulfide, Eugenol, Methyl Eugenol, Carvone, Limonene, Eucalyptol, Eudesmol, α- pinene) those were identified as major constituent compounds of the effective EOs were individually bioassayed against the larval stages of *A*. *aegypti*. The highest larvicidal potency was recorded for the compound Eudesmol with LC50 value of 2.25 ppm after 24 h exposure period. Potential larvicidal effect was also found for the compound Diallyldisulfide and Diallyltrisulfide having median sub-lethal doses ranging between 10–20 ppm. Again, moderate larvicidal activities were observed for the compound Eugenol, Limonene and Eucalyptol with LC50 value of 63.35 ppm, 139.29 ppm and 181.33 ppm at 24 h respectively (Table 3). However, Methyl Eugenol and Carvone were not found to have much larvicidal potential even at the highest dose applied and hence LC50 value was not calculated (Table 3). The synthetic larvicide Temephos showed 0.43 ppm median lethal concentration at 24 h of exposure period (Table 3, Supplementary Table 6) against *Aedes aegypti*.

**Table 3:** Sub-lethal concentrations (LC50) of different terpene compounds against 4^th^ instar larvae and adults of *Aedes aegypti*.

Sl no	Compounds	Bioassay	Time (hour)	LC50 value (ppm)	Regression equation	95%confidence level		χ^2^ value
						Lower bound	Upper bound	
1	Diallyldisulfide	Larvicidal	24	16.29	Y = 1.61 + 2.80x	2.934	4.161	7.381
			48	14.86	Y = 1.65 + 2.86x	3.248	4.730	8.112
			72	14.65	Y = 1.70 + 2.83x	3.177	4.597	8.480
		Adulticidal	24	166.02	Y = 0.38 + 2.06x	1.892	2.853	11.805
			48	94.86	Y = 0.91 + 2.07x	1.644	2.384	135.446
			72	49.05	Y = 1.63 + 1.99x	1.588	2.285	40.536
2	Diallyltrisulfide	Larvicidal	24	10.53	Y = 2.30 + 2.64x	3.805	4.452	13.148
			48	9.87	Y = 2.41 + 2.60x	3.111	4.489	10.225
			72	8.40	Y = 2.72 + 2.47	3.051	4.378	9.671
		Adulticidal	24	298.07	Y = 0.20 + 2.01x	2.096	3.228	21.587
			48	123.08	Y = 0.92 + 1.95x	1.510	2.213	18.586
			72	90.30	Y = 1.19 + 1.95x	1.543	2.243	34.429
3	Eugenol	Larvicidal	24	63.35	Y = 1.28 + 3.49x	2.705	3.952	19.145
			48	49.78	Y = 0.29 + 2.77x	2.121	3.121	21.841
			72	45.61	Y = 1.13 + 2.33x	1.915	2.760	26.050
4	Methyl Eugenol	Larvicidal	24	—	—	—	—	—
			48	—	—	—	—	—
			72	—	—	—	—	—
5	Eucalyptol	Larvicidal	24	181.33	Y = 0.12 + 2.16x	2.259	3.010	153.446
			48	138.38	Y = 0.10 + 2.29x	2.233	2.968	90.724
			72	104.50	Y = 0.39 + 2.28x	2.074	2.757	33.485
		Adulticidal	24	17.60	Y = 1.95 + 2.45x	1.910	2.897	18.049
			48	15.19	Y = 2.15 + 2.41x	1.947	2.956	18.147
			72	14.82	Y = 2.19 + 2.40x	1.957	2.972	18.160
6	Eudesmol	Larvicidal	24	2.25	Y = 3.70 + 3.68x	1.962	2.490	62.936
			48	1.91	Y = 3.62 + 4.92x	2.931	3.865	27.874
			72	1.69	Y = 3.91 + 4.76	3.048	4.030	49.381
		Adulticidal	24	1.82	Y = 3.67 + 5.13x	2.980	5.279	7.941
			48	1.14	Y = 4.75 + 4.31x	2.634	4.533	23.471
			72	1.0	Y = 4.98 + 4.29x	3.503	6.075	14.124
7	Limonene	Larvicidal	24	139.29	Y = 0.57 + 2.06x	1.719	2.409	24.405
			48	88.67	Y = 1.13 + 1.99x	1.658	2.317	23.253
			72	71.59	Y = 0.71 + 2.31x	1.891	2.673	18.866
		Adulticidal	24	737.01	Y = 2.67 + 2.67x	2.689	5.189	12.246
			48	576.77	Y = 3.43 + 3.05x	3.415	6.180	10.451
			72	435.93	Y = 3.06 + 3.05x	2.915	5.040	28.428
8	Carvone	Larvicidal	24	—	—	—	—	—
			48	—	—	—	—	—
			72	—	—	—	—	—
		Adulticidal	24	140.79	Y = 0.13 + 2.27x	1.743	2.829	7.492
			48	109.52	Y = 0.13 + 2.39x	1.826	2.971	17.575
			72	88.74	Y = 0.16 + 2.48x	1.889	3.093	22.528
9	Temephos	Larvicidal	24	0.43	Y = 5.59 + 1.60x	1.267	1.909	19.253
			48	0.34	Y = 5.79 + 1.67x	1.271	1.876	17.810
			72	0.23	Y = 6.03 + 1.61x	1.275	2.041	18.309
10	Malathion	Adulticidal	24	5.44	Y = 2.80 + 2.98x	2.108	3.582	6.381
			48	3.81	Y = 3.09 + 3.29x	2.647	4.819	9.180
			72	3.25	Y = 3.3 + 3.32x	2.857	5.092	10.498

#### Adulticidal activity

Seven compounds (Diallyldisulfide, Diallyltrisulfide, Eucalyptol, α- pinene, Eudesmol, Limonene and Carvone) those were identified as major compounds of effective EOs were tested individually against adult *Aedes aegypti*. From probit regression analysis, Eudesmol was found as the most potential with LC50 value of 1.82 ppm followed by Eucalyptol with LC50 value of 17.60 ppm at 24 h exposure time. Other five tested compounds were found to have moderate adulticidal effects with LC50 laid between 140.79 ppm to 737.01 ppm (Table 3). The efficacy of the synthetic organophosphate Malathion showed lower adulticidal effects than Eudesmol and higher than other six compounds with LC50 value of 5.44 ppm at 24 h exposure period (Table 3, Supplementary Table 6).

### Formulation of effective combination

#### Acute larvicidal effects of binary mixtures

Seven effective major compounds along with the organophosphate Temephos were selected for preparing binary combinations at their LC50 dose at 1:1 ratio. All total 28 binary combinations were prepared and tested for their larvicidal efficacy against *A*. *aegypti*. Of them nine combinations were found synergistic, fourteen combinations as antagonistic and five combinations were found to have no larvicidal effect. Among the synergistic combinations, the combination between Diallyldisulfide and Temephos was found as the most effective with 100% observed mortality at 24 hours (Table 4). Again, mixture of Limonene with Diallyldisulfide, Eugenol with Temephos showed good potentiality with 98.3% observed larval mortality, (Table 5). Other 4 combinations i.e. Eudesmol plus Eucalyptol, Eudesmol plus Limonene, Eucalyptol plus α- pinene, α- pinene plus Temephos also showed remarkable larvicidal efficacy with more than 90% observed mortality against almost 60–75% expected mortality (Table 4). However, combination of Limonene with α-pinene or Eucalyptol showed antagonistic response. Similarly, mixtures of Temephos with Eugenol or Eucalyptol or Eudesmol or Diallyltrisulfide were found antagonistic. Again, combination between Diallyldisulfide and Diallyltrisulfide and combination of anyone of these compounds with Eudesmol or Eugenol were antagonistic in larvicidal action. Combinations of Eudesmol with Eugenol or α-pinene were also recorded antagonistic.

**Table 4:** Acute Effects of Binary Mixtures (1:1) of LC50 of selected terpene compounds against fourth-instar larvae of *Aedes aegypti* and type of interactions.

Sl. no	Compound A	Compound B	% mortality at LC50 (A)	% mortality at LC50 (B)	Expected mortality in binary mixture	Observed mortality in binary mixture	χ^2^	Effect
1	Diallyldisulfide	Diallyltrisulfide	40	43.3	65.9	3.3	59.46	Antagonistic
2	Diallyldisulfide	Eudesmol	40	55	73	3.3	66.55	Antagonistic
3	Diallyldisulfide	Eucalyptol	40	41.6	64.9	86.6	7.25	Synergistic
4	Diallyldisulfide	Eugenol	40	48.3	68.9	10	50.35	Antagonistic
5	Diallyldisulfide	Limonene	40	40	64	98.3	18.38	Synergistic
6	Diallyldisulfide	α- pinene	40	46.67	67.6	63.33	0.27	No effect
7	Diallyldisulfide	Temephos	40	45	67	100	16.26	Synergistic
8	Diallyltrisulfide	Eudesmol	43.3	55	74.5	1.6	71.33	Antagonistic
9	Diallyltrisulfide	Eucalyptol	43.3	41.6	66.8	81.6	3.28	No effect
10	Diallyltrisulfide	Eugenol	43.3	48.3	70.7	6.66	58	Antagonistic
11	Diallyltrisulfide	Limonene	43.3	40	65.9	80	3.01	No effect
12	Diallyltrisulfide	α- pinene	43.3	46.67	69.22	66.67	0.094	No effect
13	Diallyltrisulfide	Temephos	43.3	45	68.8	8.3	53.20	Antagonistic
14	Eudesmol	Eucalyptol	55	41.6	73.7	95	6.16	Synergistic
15	Eudesmol	Eugenol	55	48.3	76.7	11.6	55.25	Antagonistic
16	Eudesmol	Limonene	55	40	73	90	3.96	Synergistic
17	Eudesmol	α- pinene	55	46.67	75.7	0	75.7	Antagonistic
18	Eudesmol	Temephos	55	45	75.3	1.6	72.13	Antagonistic
19	Eucalyptol	Eugenol	41.6	48.3	69.8	66.6	14.67	Antagonistic
20	Eucalyptol	Limonene	41.6	40	64.9	20	31.06	Antagonistic
21	Eucalyptol	α- pinene	41.6	46.67	68.14	96.67	11.95	Synergistic
22	Eucalyptol	Temephos	41.6	45	67.9	50	4.72	Antagonistic
23	Eugenol	Limonene	48.3	40	68.9	98.3	12.55	Synergistic
24	Eugenol	α- pinene	48.3	46.67	71.92	73.33	0.027	No effect
25	Eugenol	Temephos	48.3	45	71.5	0	71.6	Antagonistic
26	Limonene	α- pinene	40	46.67	67.6	33.33	17.37	Antagonistic
27	Limonene	Temephos	40	45	67	98.3	14.62	Synergistic
28	α- pinene	Temephos	46.67	45	70.3	96.67	9.89	Synergistic

**Table 5:** Acute Effects of Binary Mixtures (1:1) of LC50 of selected terpene compounds against third to fourth day old adult *Aedes aegypti* and type of interactions.

Sl. no	Compound A	Compound B	% mortality at LC50 (A)	% mortality at LC50 (B)	Expected mortality in binary mixture	Observed mortality in binary mixture	χ^2^	Effect
1	α- pinene	Diallyldisulfide	43.3	33.33	61.8	76.67	3.57	No effect
2	α- pinene	Diallyltrisulfide	43.3	53.33	73.2	86.67	2.47	No effect
3	α- pinene	Eucalyptol	43.3	56.67	74.9	63.33	1.79	No effect
4	α- pinene	Eudesmol	43.3	43.33	67.5	33.33	17.29	Antagonistic
5	α- pinene	Carvone	43.3	43.33	67.5	66.67	0.01	No effect
6	α- pinene	Limonene	43.3	53.33	73.2	3.3	66.75	Antagonistic
7	α- pinene	Malathion	43.3	33.33	61.8	96.67	19.67	Synergistic
8	Diallyldisulfide	Diallyltrisulfide	33.33	53.33	68.5	6.6	55.9	Antagonistic
9	Diallyldisulfide	Eucalyptol	33.33	56.67	70.5	63.23	74.9	Antagonistic
10	Diallyldisulfide	Eudesmol	33.33	43.33	61.8	23.33	23.95	Antagonistic
11	Diallyldisulfide	Carvone	33.33	43.33	61.8	3.3	55.38	Antagonistic
12	Diallyldisulfide	Limonene	33.33	53.33	68.5	10	49.95	Antagonistic
13	Diallyldisulfide	Malathion	33.33	33.33	55.1	80	11.25	Synergistic
14	Diallyltrisulfide	Eucalyptol	53.33	56.67	79.3	46.67	13.43	Antagonistic
15	Diallyltrisulfide	Eudesmol	53.33	43.33	73.2	23.33	33.98	Antagonistic
16	Diallyltrisulfide	Carvone	53.33	43.33	73.2	3.3	66.75	Antagonistic
17	Diallyltrisulfide	Limonene	53.33	53.33	77.9	6.6	65.26	Antagonistic
18	Diallyltrisulfide	Malathion	53.33	33.33	68.5	83.33	3.21	No effect
19	Eucalyptol	Eudesmol	43.33	20	54.40	76	8.57	Synergistic
20	Eucalyptol	Carvone	56.67	43.33	74.9	16.67	45.27	Antagonistic
21	Eucalyptol	Limonene	56.67	53.33	79.3	50	10.83	Antagonistic
22	Eucalyptol	Malathion	56.67	33.33	70.5	96.67	9.71	Synergistic
23	Eudesmol	Carvone	43.33	43.33	67.5	56.67	1.74	No effect
24	Eudesmol	Limonene	43.33	53.33	73.2	3.3	66.75	Antagonistic
25	Eudesmol	Malathion	43.33	33.33	61.8	100	23.61	Synergistic
26	Carvone	Limonene	23.33	26.67	43.02	100	74.99	Synergistic
27	Carvone	Malathion	43.33	33.33	61.8	96.67	19.67	Synergistic
28	Limonene	Malathion	53.33	33.33	68.51	3.3	62.06	Antagonistic

#### Acute adulticidal effects of binary mixtures

Among all the 28 binary mixtures tested for adulticidal activity, seven combinations were found to have synergistic actions, six with no effect while other fifteen were recorded with antagonistic effect. The mixture of Eudesmol plus Eucalyptol and Limonene plus Carvone were found more effective with 76% and 100% observed mortality respectively after 24 h than other synergistic combinations (Table 5). Malathion was observed to show synergistic action in combination with all the compounds excepts with Limonene and Diallyltrisulfide. On the other hand, combination between Diallyldisulfide and Diallyltrisulfide and any one of them with Eucalyptol or Eudesmol or Carvone or Limonene were found antagonistic. Similarly combination of α-pinene with Eudesmol or Limonene, Eucalyptol with Carvone or Limonene, Limonene with Eudesmol or Malathion showed antagonistic larvicidal effects. For other six combinations, the expected and observed mortalities were not found to be significantly different (Table 5).

#### Bioassay of effective combinations in large insect mass

Based on synergistic effects and sub- lethal doses, finally four combinations (Eudesmol plus Limonene, Eugenol plus Limonene, Diallyldisulfide plus Limonene and Diallyldisulfide plus Temephos) were selected and further tested for their larvicidal toxicity against large numbers of *Aedes aegypti*. The results showed 100% observed larval mortalities in response to binary combinations of Eugenol–Limonene, Diallyldisulfide-Limonene, Diallyldisulfide–Temephos against 76.48%, 72.16% and 63.4% expected larval mortalities respectively (Table 6). The combination between Limonene and Eudesmol was comparatively less effective showing 88% observed larval mortality at 24 h exposure period (Table 6). So, in large scale application too, the selected four binary combinations showed synergistic larvicidal effect against *A*. *aegypti* (Table 6).

**Table 6:** Acute effects of Binary mixtures (1:1) of LC50 dose of selected terpene compounds against 4^th^ instar larvae and adults of *Aedes aegypti* and type of interaction after large scale application (n = 300 for larva and 150 for adult).

Bioassay	Compound A	Compound B	% mortality at LC50 (A)	% mortality at LC50 (B)	Expected mortality in binary mixture	Observed mortality in binary mixture	χ^2^	Effect
Larvicidal	Eudesmol	Limonene	27.66	52	64.96	87.66	7.93	Synergistic
	Eugenol	Limonene	51.33	52	76.48	100	7.23	Synergistic
	Diallyldisulfide	Limonene	42.66	52	72.16	100	10.74	Synergistic
	Diallyldisulfide	Temephos	42.66	36.67	63.46	100	21.04	Synergistic
Adulticidal	Eudesmol	Eucalyptol	33	41	60.46	78.66	5.47	Synergistic
	Carvone	Limonene	44	33.66	65.84	100	17.72	Synergistic
	Eudesmol	Malathion	33	43.66	61.81	100	23.59	Synergistic

For adulticidal bioassay, three synergistic combinations were selected to be applied against large numbers of adult *A*. *aegypti*. For selection of combinations to be tested against large insect mass, at first emphasis was given on the best two synergistic combinations of terpene compounds that was combination of Carvone plus Limonene and Eucalyptol plus Eudesmol. Secondly one best synergistic combination was selected from the pair of synthetic organophosphate Malathion with terpene compound. Here we consider the combination of Malathion plus Eudesmol as the best combination to be tested against large insect mass because of its maximum observed mortality and the very low LC50 values of the constituent candidates. Malathion showed synergistic action while combined with α-pinene, Diallydisulfide, Eucalyptol, Carvone and Eudesmol. But if we look at the LC50 values, the value for Eudesmol was the lowest (2.25 ppm). The calculated LC50 values for Malathion, α-pinene, Diallydisulfide, Eucalyptol, Carvone were 5.4, 716.55, 166.02, 17.6, 140.79 ppm respectively. These values indicated that the combination between Malathion and Eudesmol as the best combination from the dose point of view. The results revealed that the combination of Carvone plus Limonene and Eudesmol plus Malathion showed 100% observed mortality against 61% to 65% expected mortalities. Another combination, Eudesmol plus Eucalyptol showed 78.66% mortality against 60% expected mortality after 24 h exposure period. All the three selected combinations showed synergistic action in combination even in large-scale applications against adults of *Aedes aegypti* (Table 6).

## Discussion

In the present investigation selected plant EOs of Mp, As, Os, Em and Cl showed promising lethal effects against larval and adult stages of *Aedes aegypti*. Larvicidal activity of EO of Mp was recorded highest with LC50 value of 0.42 ppm followed by EOs of As, Os and Em having LC50 value below 50 ppm at 24 h. These findings were in line with the previous studies carried on mosquitoes and another dipteran flies^10–14^. Although larvicidal potency of Cl was comparatively lower with LC50 value of 163.65 ppm at 24 h than other EOs, its adulticidal potential was found highest having LC50 value of 23.37 ppm at 24 h. EOs of Mp, As and Em also showed good adulticidal potential having LC50 value within the range of 100–120 ppm at 24 h exposure period but comparatively lower than their larvicidal efficiency. On the other hand, EO of Os showed negligible adulticidal effect even at the highest dose of treatment. Thus, the result reflects that the toxicity of the plant EOs may vary with respect to developmental stages of the mosquito^15^. It also depends on penetration rate of EO into the insect body, their interaction with specific target enzymes and detoxification ability of the mosquito at each developmental stage^16^. A good number of studies indicate that the major constituent compound(s) are responsible factor for bioactivity of an EO as it comprises the major fraction of the total compounds^3,12,17,18^. Therefore, we considered two major compounds from each EO. From GC-MS result Diallyldisulfide and Diallyltrisulfide were identified as the major compounds of EO of As which was in conformity with the previous reports^19–21^. Again, Carvone and Limonene were identified as the major compounds of EO of Mp although the previous report suggested menthol as one of its major compound^22,23^. Constituent profile of the EO of Os revealed Eugenol and Methyl Eugenol as the major compounds showing similarity with the findings of earlier researchers^16,24^. Eucalyptol and Eudesmol were recorded as principal compounds present in Em leaf oil which was in line with the findings of some researchers^25,26^ but contradicting the findings of Olalade *et al*.^27^. Dominance of Eucalyptol and α- pinene was observed in EO of *Callistemon linearis* showing similarity with previous studies^28,29^. Whatever intraspecific variation for constituent composition and concentration of EO extracted from the same plant species from different places has been reported and also observed in the present study were influenced by geographical conditions where the plant grows, harvesting time, development stage or age of the plant and occurrence of chemotypes etc.^22,30–32^. The identified major compounds were then purchased and tested for their larvicidal and adulticidal effects against *Aedes aegypti*. The result revealed that the larvicidal activity of Diallyldisulfide was equal to the activity of crude EO of As. But the activity of Diallyltrisulfide was higher than EO of As. These findings were similar to the findings of Kimbaris *et al*.^33^ working on *Culex pipines*. However, these two compounds did not show good adulticidal activity against the target mosquito which was in conformity with the findings of Plata- Rueda *et al*.^34^ worked on *Tenebrio molitor*. EO of Os was found effective against larval stages of *Aedes aegypti* but not against adult stages. The larvicidal activity of major individual compound was found lesser than the activity of crude EO of Os. This implies the role of other compounds and their interactions in crude EO. Methyl Eugenol individually possessed negligible activity whereas Eugenol individually possessed moderate larvicidal activity. This finding one-way supported^35,36^ and other way contradicted the findings of earlier investigators^37,38^. The difference in the functional group between Eugenol and Methyl Eugenol might make the differences of their toxicity against the same target insect^39^. Limonene was found to possess moderate larvicidal activity while the Carvone showed negligible effect. Similarly, comparatively lower toxicity of Limonene and higher toxicity of Carvone against adults supported the findings of some previous studies^40^ and opposed others^41^. Possession of double bond both in endocyclic and exocyclic position might add advantage of these compounds as larvicidal agent^3,41^ while as a ketone Carvone with unsaturated α and β carbon might show higher toxicity as adulticides^42^. However, individual performance of Limonene and Carvone were quite lower than the whole EO of Mp (Tables 1, 3). Among the terpene compounds tested, Eudesmol was found to possess highest effects both as larvicide and adulticide with LC50 value below 2.5 ppm and emerged as promising compound for *Aedes* control. Its performance was better than whole EO of Em though it was not in line with the finding of Cheng *et al*.^40^. Eudesmol, a sesquiterpene with two isoprene units are less volatile than oxygenated monoterpene like Eucalyptoland thus have a greater potential as insecticide. Eucalyptol by its own showed higher adulticidal than larvicidal activity and both supported and opposed by the findings of earlier workers^37,43,44^. The individual activity was almost at par with the activity of whole EO of Cl. Another bicyclic monoterpene, α- pinene was found to possess lower adulticidal effect than larvicidal effect against *Aedes aegypti* which was opposite to the performance of whole EO of Cl. Overall insecticidal activity of a terpene compound is affected by its lipophilicity, volatility, branching of the carbon-atom, projection area, surface area, functional group and their position etc.^45,46^. The compounds may exert effects by disintegrating cell mass, blocking respiratory activity, interrupting nerve impulse transmissionetc^47^. The larvicidal activity of synthetic organophosphate-Temephos was found to have highest effect with LC50 value of 0.43 ppm, which was in accordance with the findings of Lek- Uthal^48^. The adulticidal activity of synthetic organophosphate -Malathion was recorded as 5.44 ppm. Although, both the organophosphates showed good response against the laboratory strain of *Aedes aegypti*, development of resistance by mosquitoes against these compounds have been reported from different parts of the globe^49^. However, no such reports of resistance development were found to documentagainst botanicals^50^. Therefore, botanicals are considered as potential alternative to chemical insecticide in vector control programme.

Out of 28 binary combinations (1:1) prepared from effective terpene compounds and terpene compound with Temephos to test for larvicidal action, nine combinations were found to have synergistic, 14 combinations with antagonistic and five combinations were found to have no effect. On the other hand, in case of adulticidal bioassay, seven combinations were found to have synergistic, 15 combinations antagonistic, while six combinations were recorded to have no effect. The reason for synergistic action of some combinations might be due to the interactions of candidate compounds on different vital pathways at a time or due to the serial inhibition of different key enzymes of a particular biological pathway^51^. Combinations of Limonene with Diallyldisulfide or Eudesmol or Eugenol were found synergistic both in small scale and large-scale application (Table 6) while its combination with Eucalyptol or α-pinene was found to show antagonistic effect against larvae. On an average Limonene was found as a good synergist which might due to the presence of Methyl group, good cuticular penetration and different mode of actions^52,53^. It was reported earlier that Limonene could exert its toxic effect by penetrating through insect cuticle (contact toxicity) or targeting digestive system (anti-feeding) or acting on the respiratory systems (fumigant activity)^54^ while phenylpropanoid like Eugenol might target metabolic enzymes^55^. Thus, compounds with the different mode of actions in combination might increase total lethal actions in mixtures. Eucalyptol with Diallyldisulfide or Eudesmol or α-pinene was found synergistic but rest of the combination with other compounds were either having no larvicidal effect or having antagonistic action. Earlier studies demonstrated inhibitory activity of Eucalyptol on AChE as well as octapamine and GABA receptors^56^. As cyclic monoterpenes, Eucalyptol, Eugenol, etc. might share the same mode of action like neurotoxic activity^57^ thereby minimizing their combined effect by inhibiting each other. Again, the combination of the Temephos with Diallyldisulfide, α-pinene and Limonene were found synergistic that supported previous reports of synergism that occurred between plant product and synthetic organophosphate^58^.

The combination between Eudesmol and Eucalyptol was found synergistic against both larval and adult stages of *Aedes aegypti* which might be due to their different mode of action because of their dissimilar chemical structures. Eudesmol, which is a sesquiterpene might target respiratory system^59^ while Eucalyptol, which is a monoterpene might affect acetylcholine esterase enzyme^60^. The combined effect of constituents on two or more target sites might boost total lethal actions of the combination. In case of adulticidal bioassay, Malathion was found to show synergism with Carvone or Eudesmol or Eucalyptol or Diallylldisulfide or α-pinene reflecting it as a good synergistic adulticidal candidate for combination with the entire terpene compounds except Limonene and Diallyltrisulfide. Similar finding of synergism of Malathion with plant extracts was reported by Thangam and Kathiresan^61^. This synergistic response might due to the combined toxic effects of Malathion and phytochemicals on detoxifying enzymes of insect body. Organophosphate like Malathion generally exerts their effect by inhibiting esterases, cytochrome P450 monooxygenase enzymes^62–64^. Therefore, combination of Malathion having these modes of action with terpene compounds having different mode of actions might enhance the total lethal effect against the mosquito.

On the other hand, antagonistic effect indicates that the selected compounds in a combination are less active than the individual effect of each compound. The reason for antagonism in some combinations might due to the alternation of behavior of one compound by the other compound by changing the rate of absorption, distribution, metabolism or excretion as has been suggested as possible mechanism of antagonism in combination of drug molecules by earlier researchers^65^. Again, the possible cause of antagonism might be due to the competition of constituent compounds for single receptor or target site due to similar mode of action. In some cases, non-competitive inhibition of target protein might also occur. In the present study the two organosulfur compounds namely Diallyldisulfide and Diallyltrisulfide showed antagonism possibly for the competition for the same target site. Again, these two sulfur compounds while combined with Eudesmol and α-pinene showed antagonistic and no effect. Eudesmol and α-pinene are cyclic in nature while Diallyldisulfide and Diallyltrisulfide are aliphatic in nature. Based on the chemical structure the total lethal activity supposed to enhance in combination of these compounds as their target sites are usually different^34,47^ but experimentally we found antagonistic effect which might be due to some unknown biological interactions of these compounds in living system. Similarly, combination of eucalyptol with α-pinene resulted antagonistic response though the target site of action of these two compounds were reported differently by earlier researchers^47,60^. As both of the compounds are cyclic monoterpene, there may have certain common target site to which they might compete for binding and influenced overall toxicity of the combination pair in the study.

Considering LC50 values and observed mortalities, the two best synergistic combinations of terpene compounds viz.pair of Carvone plus Limonene and Eucalyptol plus Eudesmol and one best synergistic combination of the synthetic organophosphate Malathion with terpene compound i.e. Malathion plus Eudesmol were chosen for adulticidal bioassay to test against large insect mass to confirm whether these effective combinations would work against large number of individuals in comparatively larger exposure space. All these combinations showed synergistic response against large insect mass. Similar results were found for the best larvicidal synergistic combinations which were tested against large numbers of *A. aegypti* larvae. Therefore, it can be stated that the effective synergistic larvicidal and adulticidal combinations of plant based EO compounds are competent candidate against existing synthetic chemicals and can further be used to control *Aedes aegypti* population. Similarly, the effective combination of synthetic larvicide or adulticide with terpene compounds may further be used to reduce the dose of Temephos or Malathion to be applied against the mosquito. These synergistic combinations with potent efficacy may offer solution to check resistance evolution in *Aedes* mosquitoes in future.

## Materials and Methodology

### Establishment of *Aedes aegypti* colony

Eggs of *Aedes aegypti* was collected from Indian Council of Medical Research- Regional Medical Research Centre, Dibrugarh and reared in the Department of Zoology, Gauhati University under controlled temperature (28 ± 1 °C) and humidity (85 ± 5%) following the methods described by Arivoli *et al*.^66^. After hatching, larvae were fed with larval food (powdered dog biscuit and yeast at a ratio of 3:1) while adults were fed on 10% glucose solution. From 3^rd^ day after emergence, the adult female mosquitoes were allowed to feed on blood of albino rat. Filter paper submerged in water kept in beaker was put inside the cage for egg laying.

### Collection of plant materials and EO extraction

Selected plant samples i.e. leaves of *Eucalyptus maculata* (Family- Myrtaceae), *Ocimum sanctum* (Family-Lamiaceae), *Mentha piperita* (Family- Lamiaceae), *Callistemon linearis*(Family- Myrtaceae) and bulbs of *Allium sativum* (Family-Amaryllidaceae) were collected from Guwahati and identified at Department of Botany, Gauhati University. Collected plant samples (500 g) were hydrodistilled using Clevenger apparatus for 6 hours. Extracted EOs were collected in clean glass vials and stored at 4 °C for further study.

### Bioassay of essential oils

#### Larvicidal assay

The standard WHO procedure^67^ was used with slight modification to investigate the larvicidal toxicity. DMSO was used as emulsifying agent. Initially 100 and 1000 ppm concentration of each EO was tested exposing 20 larvae per replication. Based on the result, a series of concentration was applied and the mortality was recorded from 1 hour to 6 hours at one-hour time interval and at 24-hour, 48 hour and 72 hours after treatment. The sub-lethal (LC50) concentration was determined after 24, 48- and 72-hour of exposure period. Each concentration was assayed in triplicate along with one negative control (water only) and one positive control (DMSO treated water). If the pupation occurred and more than 10% larvae died in the control group then the test was repeated. If mortality occurred in the control groups between 5–10% then, then Abbot’s correction formula^68^ was used.

#### Adulticidal assay

The method described by Ramar *et al*.^69^ was followed for adulticidal bioassay against *Aedes aegypti*, where acetone was used as a solvent. Initially, 100 and 1000 ppm concentration of each EO were tested against adult *Aedes aegypti*. 2 ml of each prepared solution was applied on Whatman no. 1 filter papers (size 12 × 15 cm^2^) and allowed to evaporate acetone for 10 minutes. Filter paper treated with 2 ml of acetone alone was used as control. After evaporation of acetone, both the treated and the control filter paper were placed in cylindrical tubes (depth 10 cm). Ten numbers of 3–4 days old non-blood fed mosquitoes were transferred in each of the three replicas of each concentration. Based on the result of initial test, different concentrations of the selected oils were tested. Mortality was recorded at 1 hour, 2 hour, 3 hour, 4 hour, 5 hour, 6 hour, 24 hour, 48 hour and 72 hour respectively from the time of the mosquito released. LC50 value was calculated at 24 h, 48 h and 72 h of exposure period. If mortality exceeded 20% in the control batch, the whole test was repeated. Again, if mortality in the controls was above 5%, results with the treated samples were corrected using Abbott’s formula^68^.

### Analysis of effective essential oil components

For analysis of the constituent compounds of the selected EO, Gas chromatography (Agilent 7890A) and mass spectrometry (Accu TOF GCv, Jeol) was performed. GC was equipped with a FID detector and a capillary column (HP5- MS). The carrier gas was helium at a flow rate of 1 ml/min. The GC programme was set for *Allium sativum* as 10: 80- 1M- 8-220-5M-8-270-9M, for *Ocimum sanctum* as 10:80-3M-8-200-3M-10-275-1M-5-280, for *Mentha piperita* as 10:80-1M-8-200-5M-8-275-1M-5-280, for *Eucalyptus maculata* as 20,60-1M-10-200-3M-30-280, and for *Callistemon linearis* as 10: 60-1M-8-220-5M-8-270-3M respectively.

### Identification of major terpene compounds of different EOs

Major compounds of each EO were identified based on their area percentage calculated from the GC- chromatogram and mass spectrometry results in reference to NIST standard database^70^.

### Bioassays of individual major terpene compounds against *A*. *aegypti*

Two major compounds from each EO were chosen from GC- MS result and purchased from Sigma-Aldrich having 98–99% purity for further bioassay. Larvicidal and adulticidal efficacy of these compounds against *A*. *aegypti* was tested following the methods described above. The most commonly used synthetic commercial larvicide Temephos (Sigma Aldrich) and adulticide Malathion (Sigma Aldrich) were assayed for comparing their efficacy with selected EO compounds following the same procedure.

### Formulation of terpene compounds

Binary mixtures of selected terpene compounds and terpene compound plus commercial organophosphates (Temephos and Malathion) were prepared mixing LC50 dose of each candidate compound in 1:1 ratio. Prepared combinations were tested against both larval and adult stages of *Aedes aegypti* following the method described above. Each bioassay was performed in triplicate for each combination and three replicates for the individual compounds present in the respective combination. Mortalities of the target insect were recorded at 24 hours. Expected mortality of the binary mixtures was calculated based on following formula.

$${\rm{E}}={{\rm{O}}}_{{\rm{a}}}+{{\rm{O}}}_{{\rm{b}}}({\rm{1}}-{{\rm{O}}}_{{\rm{a}}})$$

where, E = Expected mortality of *A*. *aegypti* in response to binary combination i.e. in Compound (A + B).

O_a_ = Observed mortalities of *A*. *aegypti* in response to the compound A at LC50 dose.

O_b_ = Observed mortalities of *A*. *aegypti* in response to the compound B at LC50 dose.

The effects of each binary mixture were marked as synergistic, antagonistic and no effect based on their calculated χ^2^value following the method described by Pavela^52^. χ^2^ value was calculated for each combination using following formula.

$${{\rm{\chi }}}^{{\rm{2}}}={({{\rm{O}}}_{{\rm{m}}}-{\rm{E}})}^{{\rm{2}}}/E$$

where, O_m_ = Observed mortality of *A*. *aegypti* in response to binary mixtures.

E = Expected mortality of *A*. *aegypti* in response to binary mixtures.

The effect of a combination was designated as synergistic when the calculated χ^2^ value was found greater than the table value at respective degrees of freedom at 95% confidence interval and if observed mortality was found greater than the expected mortality. Again, if the calculated χ^2^ value for any combination was greater than the table value at definite degrees of freedom but observed mortality was found lower than the expected one, then that treatment was considered as antagonistic. While if in any combination, calculated χ^2^ value found less than the table value at respective degrees of freedom then that combination was considered to have no effect.

### Bioassay of effective combinations in large insect mass

Based on the observed mortality in binary mixtures having synergistic action and the LC50 dose of each terpene compound present in the respective mixture, three to four potential synergistic combinations were selected to test for larvicidal and adulticidal activity against large number of insects (100 larvae and 50 adults) by following the method described above. Along with the mixtures, the individual compound present in the selected mixtures were also tested against same numbers of *Aedes aegypti* larvae and adults. The proportion of the combination was one part of LC50 dose of one candidate compound and one part of LC50 dose of another constituent compound. In adulticidal bioassay, selected compounds were dissolved in acetone solvent and applied in filter paper wrapped inside a cylindrical plastic vessel with 1300 cm^3^ volume. The acetone was evaporated for 10 minutes before releasing adult insects. Again, in case of larvicidal bioassay, LC50 doses of the candidate compounds were first dissolved in equal amount of DMSO and then mixed in 1 liter of water kept in 1300 cm^3^ plastic vessel and the larvae were released.

### Statistical analysis

The mortality data recorded were subjected to probit analysis^71^ for calculating LC_50_ values using SPSS (version 16) and Minitab software.

## Supplementary information

Dataset 1
