# Supplementary material for: Combinations of Plant Essential Oil Based Terpene Compounds as Larvicidal and Adulticidal Agent against Aedes aegypti (Diptera: Culicidae)
Source: Sci Rep. 2019 Jul 1;9:9471. doi: 10.1038/s41598-019-45908-3 (PMC6602964; doi:10.1038/s41598-019-45908-3)
Supplement: Supplementary file 1 — Dataset 1 [file 41598_2019_45908_MOESM1_ESM.pdf]

Combinations of Plant Essential Oil Based Terpene Compounds as Larvicidal and Adulticidal Agent against Aedes aegypti (Diptera: Culicidae)

Riju Sarma[1], Kamal Adhikari[2,] Sudarshana Mahanta[3], Bulbuli Khanikor[4]*
[1]Department of Zoology, Gauhati University, Guwahati, Assam, India
[2]Department of Zoology, Gauhati University, Guwahati, Assam, India
[3]Department of Zoology, Gauhati University, Guwahati, Assam, India
[4]Department of Zoology, Gauhati University, Guwahati, Assam, India
*Corresponding author
E- mail: khanikorbulbuli@yahoo.co.in

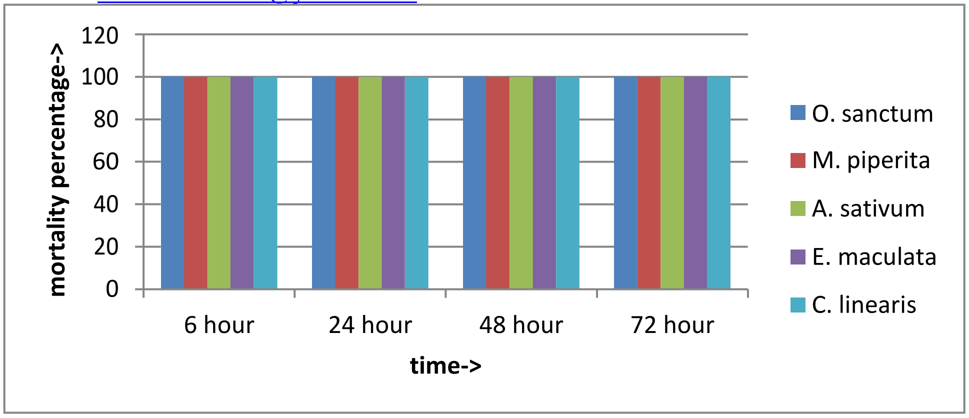

Fig 1. Mortality percentage of 4th instar larvae of *Aedes aegypti* at 1000ppm concentration of *O. sanctum, A. sativum, M. piperita*, *E. maculata and C. linearis*

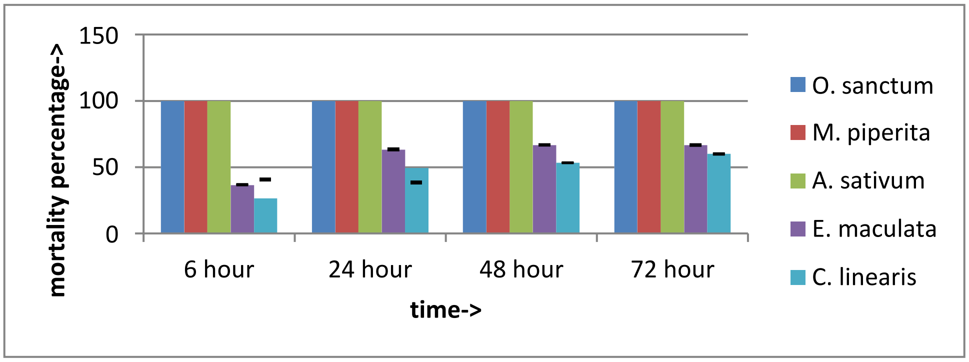

Fig 2. Mortality percentage of 4th instar larvae of *Aedes aegypti* at 100ppm concentration of *O. sanctum, A. sativum, M. piperita*, *E. maculata and C. linearis*

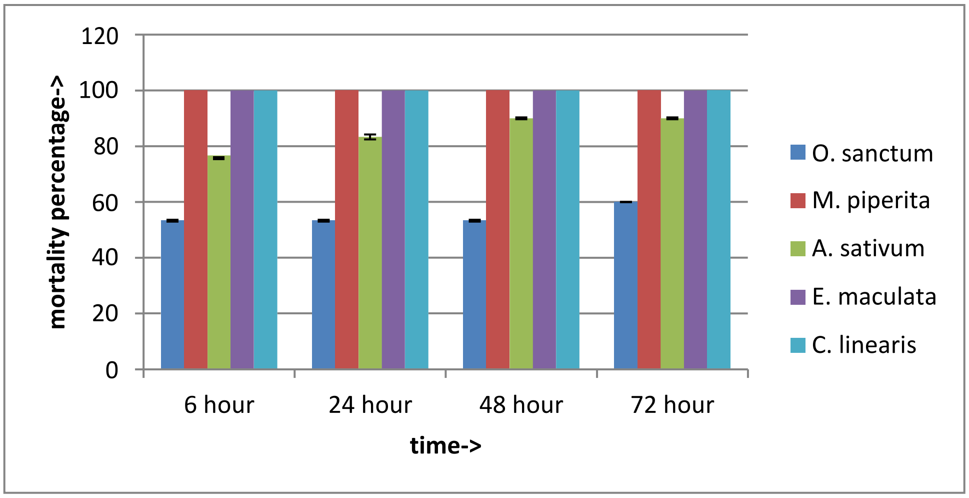

Fig 3. Mortality percentage of adult *Aedes aegypti* in response to 1000ppm concentration of *O. sanctum*, *A. sativum*, *M. piperita*, *E. maculata and C. linearis* at different time interval

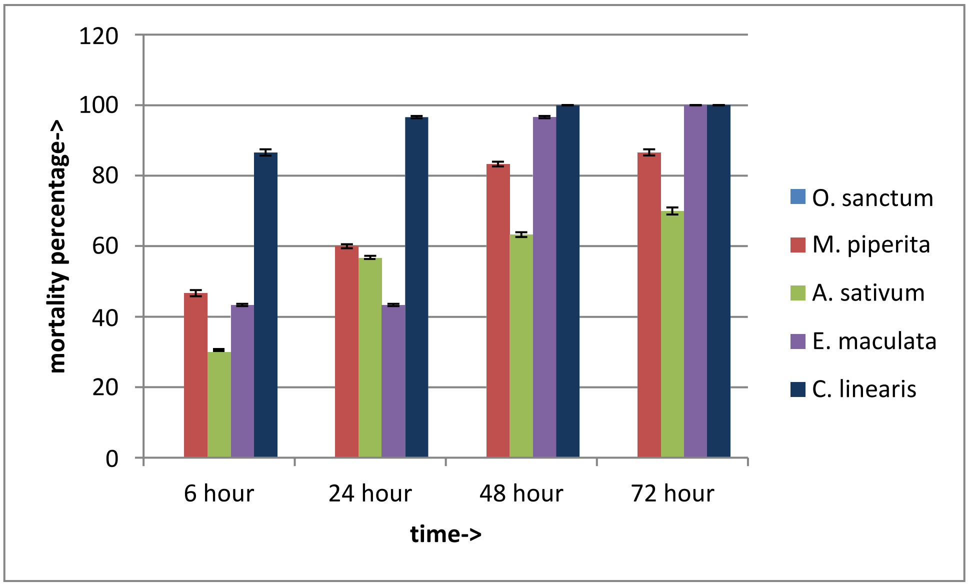

Fig 4. Mortality percentage of adult *Aedes aegypti* in response to 100ppm concentration of *O. sanctum*, *A. sativum*, *M. piperita*, *E. maculata and C. linearis* at different time interval

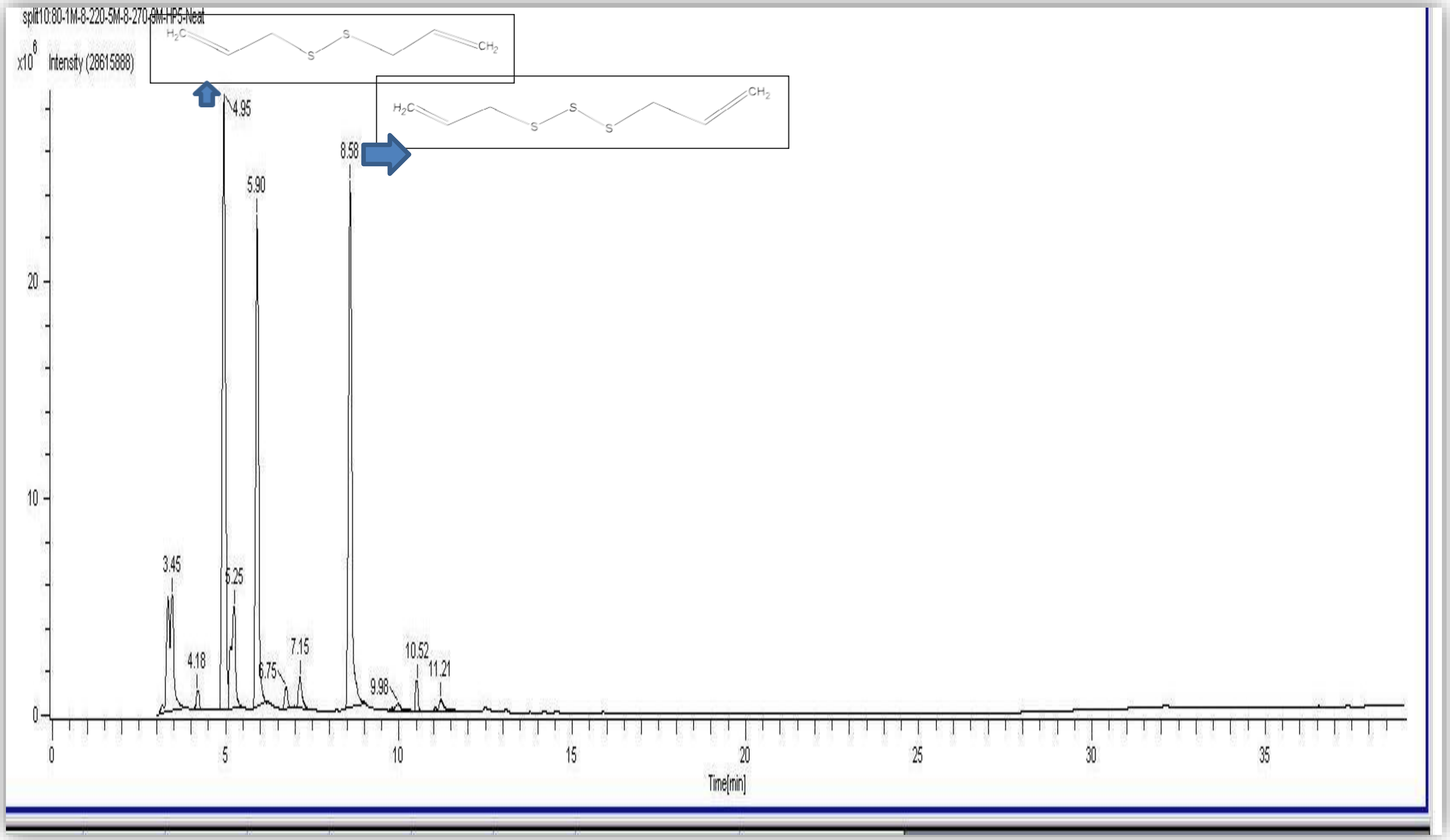

Fig 5. GC Chromatogram of the essential oil of the bulbs of *Allium sativum*

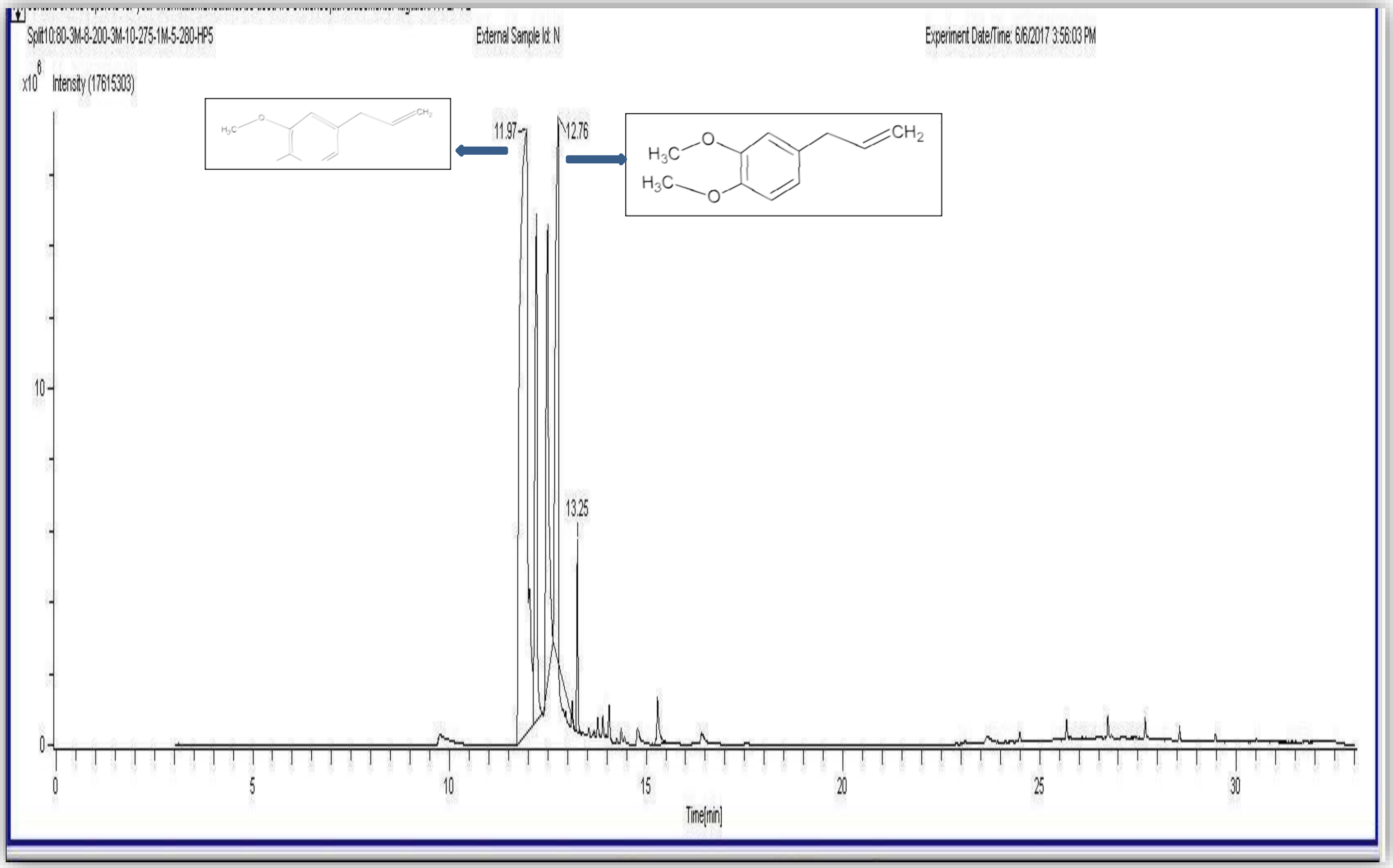

Fig 6- GC Chromatogram of the essential oil of the leaves of *Ocimum sanctum*

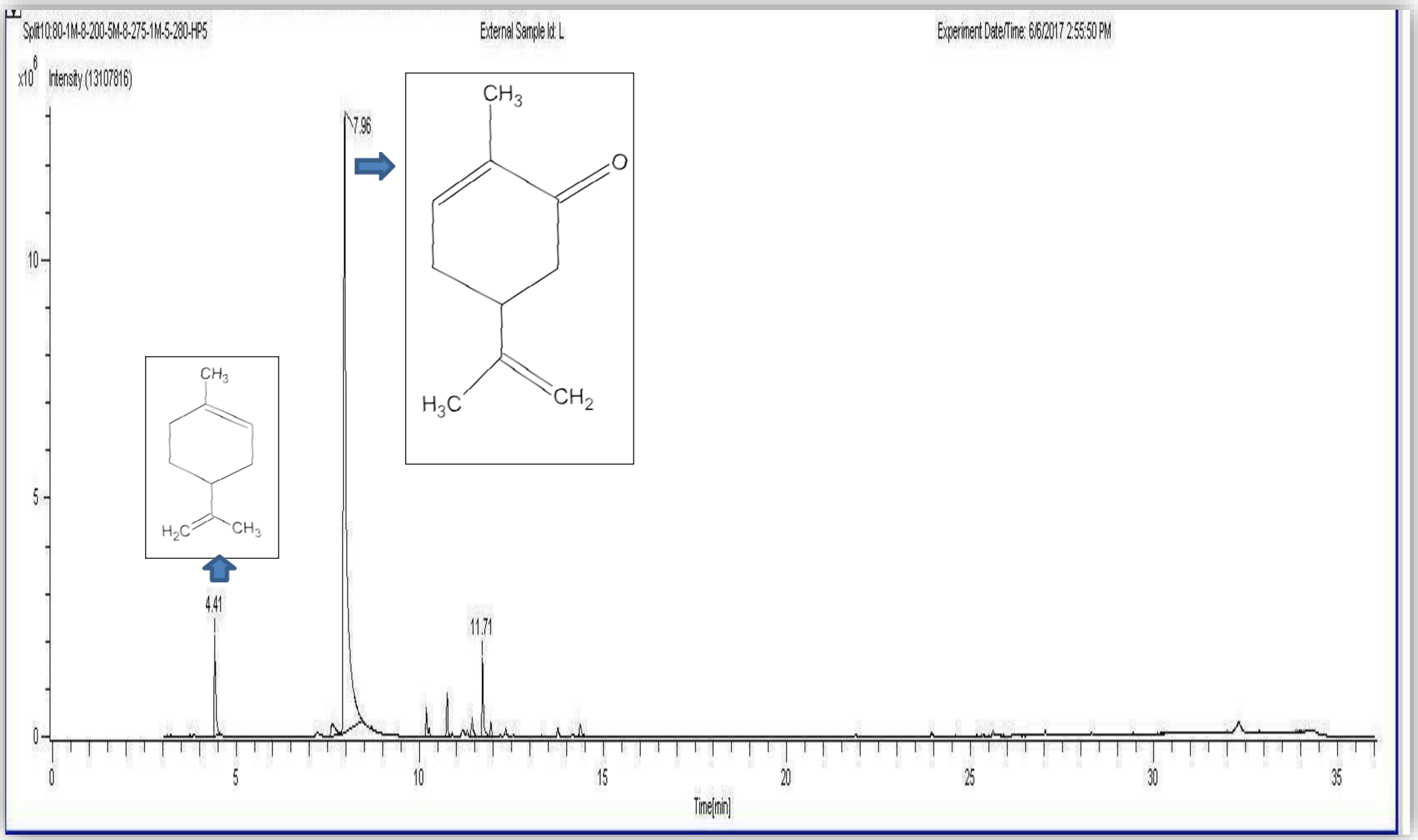

Fig 7. GC Chromatogram of the essential oil of the leaves of *Mentha piperita*

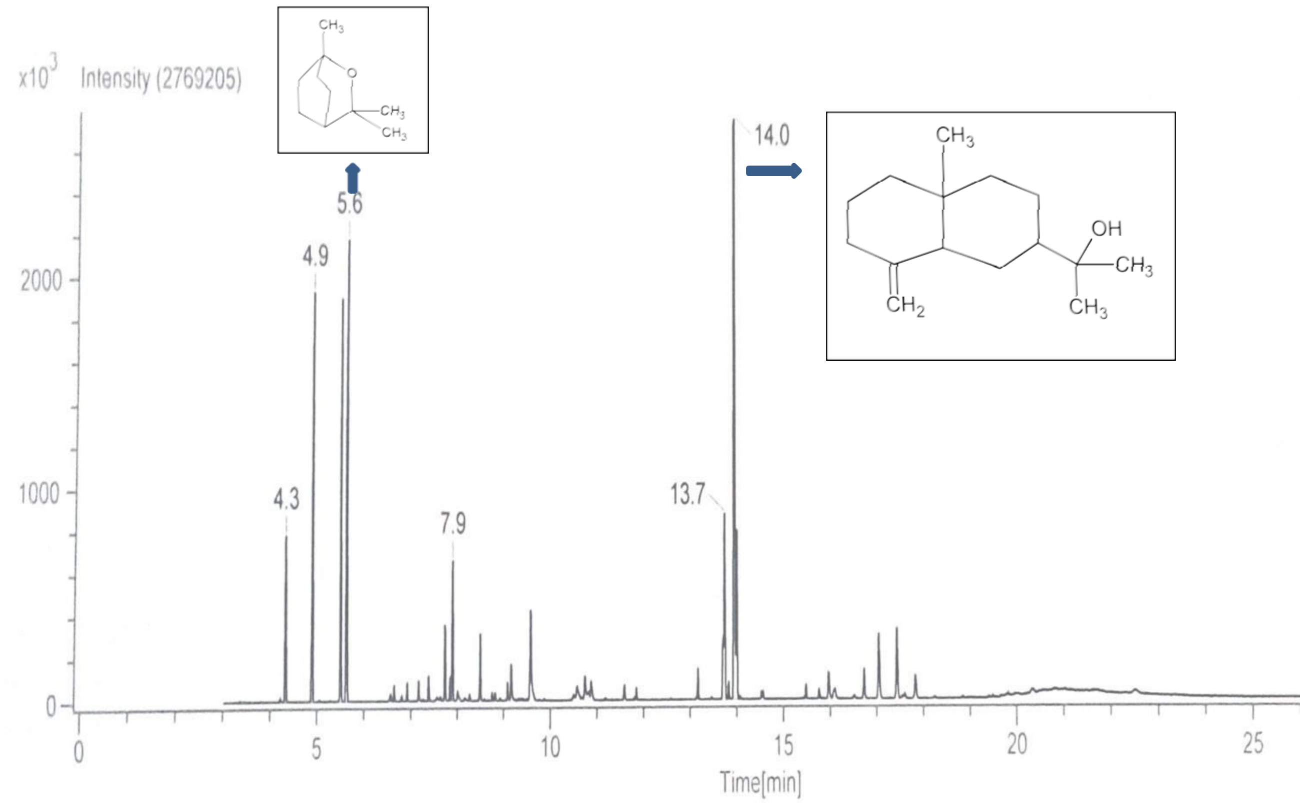

Fig 8. GC Chromatogram of the essential oil of the leaves of *Eucalyptus maculata*

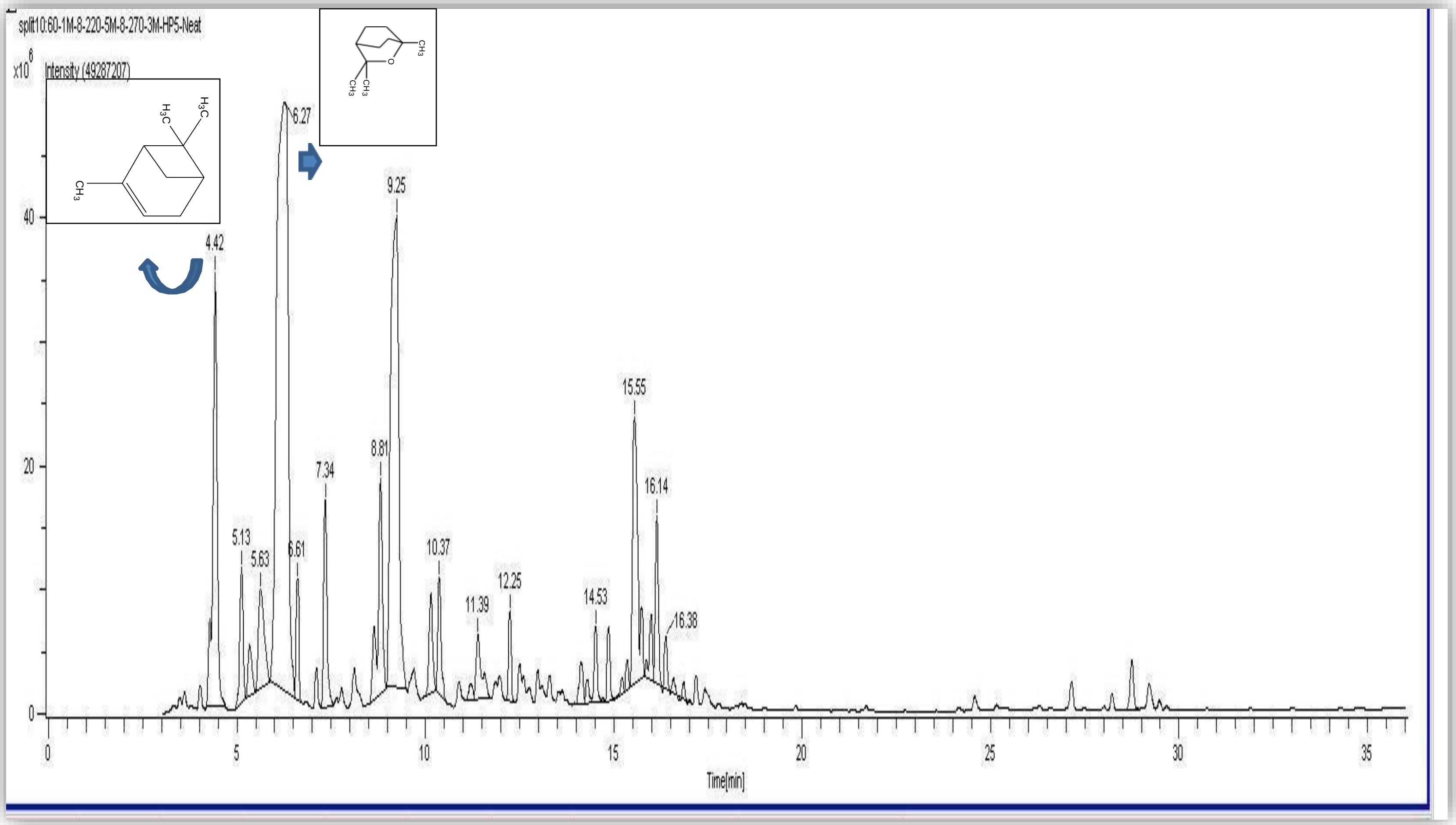

Fig 9- GC Chromatogram of the essential oil of leaves of *Callistemon linearis*

Table-1- Different constituent compounds present in the essential oil of *Allium sativum* extracted from bulb (results obtained from NIST database after GC-MS analysis)

Sl. no	Compounds	Prob (%)	Retention index	Chemical Formula	Molcular Weight	Area (%)	Retention time
1	Actinobolin	63.1	2743	$C_{13}H_{20}N_2O_6$	300	-	17.93
2	Dimethyl trisulphide	96.9	943	$C_2H_6S_3$	126	3.35	3.45
3	Eucalytol	71.1	1023	$C_{10}H_8O$	154	0.24	4.18
**4**	**Diallyl disulphide**	**98.7**	**1056**	$\mathbf{C_6H_{10}S_2}$	**146**	**8.51**	**4.95**
5	Diallyl disulphide	82.4	1056	$C_6H_{10}S_2$	146	2.14	5.25
6	Trisulphide methyl propenyl	38.5	1112	$C_4H_8S_3$	152	6.42	5.89
7	3-vineyl- 1,2 diithacyclohex-4-ene	74.7	1205	$C_6H_8S_2$	144	0.29	6.75
8	3-vineyl- 1,2 diithacyclohex-5-ene	96.8	1155	$C_6H_8S_2$	144	0.50	7.15
**9**	**Diallyltrisulphide**	**96.8**	**1283**	$\mathbf{C_6H_{10}S_3}$	**178**	**7.75**	**8.58**
10	Bicyclo(7,2,0)undec-4-ene-4,11,11-trimethyl-8-methylene	18.7	1396	$C_{15}H_{24}$	204	0.36	10.51
12	2-H-1-Benzopyran, 7-methoxy-2,2-Dimethyl	79.4	1440	$C_{12}H_{14}O_2$	190	0.35	11.21
13	2-Hydroxy-3-methoxy succinic acid, dimethyl ether	24.7	1226	$C_7H_{12}O_6$	192	-	24.7

Table-2- Different constituent compounds present in the essential oil of *Ocimum sanctum* extracted from leaves (results obtained from NIST database after GC-MS analysis)

Component	Prob(%)	Molecular weight	Retention index	Chemical formula	Area (%)	Retention time
**Eugenol**	**31.7**	**164**	**1337**	$\mathbf{C_{10}H_{12}O_2}$	**52.30**	**11.86**
Cyclohexane-1-ethelyl- 1-methyl	23.2	204	1398	$C_{15}H_{24}$	13.26	12.18
**Methyleugenol**	**83**	**178**	**1402**	$\mathbf{C_{11}H_{14}O_2}$	**14.87**	**12.48**
α- Caryophyllene	61.3	204	1456	$C_{15}H_{24}$	-	13.24

Cyclohexane-1- ethyl-1-methyl	17.7	204	1398	$C_{15}H_{24}$	1.20	14.05
Caryophyllene oxide	50.1	220	1576	$C_{15}H_{24}O$	1.26	15.30
Sulfurous acid hexyl penta decyl	6.73	376	2732	$C_{21}H_{24}O_3S$	-	25.68

Table-3- Different constituent compounds present in the essential oil of *Mentha piperita* extracted from leaves (results obtained from NIST database after GC-MS analysis)

Component	Prob (%)	Molecular weight	Retention index	Chemical formula	Area (%)	Retention time
**Limonene**	**25.8**	**136**	**1014**	**$C_{10}H_{16}$**	**6.65**	**4.43**
**Carvone**	**47.1**	**150**	**1220**	**$C_{10}H_{14}0$**	**79.6**	**7.96**
β- Boubonene	54.8	204	1408	$C_{15}H_{24}$	1.84	10.19
(+)-Epibicyclosesquiphellandrer	40.5	204	1470	$C_{15}H_{24}$	1.34	11.42
Germecene D	40.4	204	1480	$C_{15}H_{24}$	4.54	11.72
α- cardinol	69.7	222	164	$C_{15}H_{26}0$	1.03	14.37

Table-4- Different constituent compounds present in the essential oil of *Eucalyptus maculata* extracted from leaves (results obtained from NIST database after GC-MS analysis)

Sl. No	Compounds	Prob(%)	Molecular weight	Chemical formula	Area (%)	Retention time
1	α- pinene	11.7	136	$C_{10}H_{16}$	6.91	4.3
2	β-pinene	45.7	136	$C_{10}H_{16}$	15.01	4.90
3	Benzene, 1- methyl-2-(1- methylethyl)	29.9	134.22	$C_{10}H_{14}$	13.60	5.5
**4**	**Eucalyptol**	**87.5**	**154**	**$C_{10}H_{18}O$**	**17.64**	**5.6**
**5**	**Eudesmol**	**81.7**	**222.37**	**C15H26O**	**31.80**	**14**

Table-5- Different constituent compounds present in the essential oil of *Callistemon linearis* extracted from leaves (results obtained from NIST database after GC-MS analysis)

Sl. no	Compounds	Prob(%)	Molecular weight	Retention index	Chemical formula	Area (%)	Retention time
1	4,6 di-l-butylpyrogallol	18.6	238	1936	$C_{14}H_{22}O_3$	.029	17.18
2	4a, 6a- dimethyl 2-oxo-1a, 2,<br>4a, 4b,	22.1	344	2274	$C_{21}H_{28}O_4$	.86	28.27

	5,6,6a,7,8,9,9a,9b,10,11, tetra decahydrocyclopental (1,8)phenanthro (1,10a-b)oxiten-7-yl acetate pk1						
**4**	**α- pinene**	**15.7**	**136**	**931**	**$C_{10}H_{16}$**	**9.28**	**4.43**
5	β-pinene	32.5	136	970	$C_{10}H_{16}$	2.21	5.12
6	β-myrecene	36.3	136	979	$C_{10}H_{16}$	1.01	5.34
7	Bicyclo(3,1,0)hexane, 4-methyl-1-(1-methylethyl, didehydro derivative	10.6	136	873	$C_{10}H_{16}$	2.87	5.64
**8**	**Eucalyptol**	**67.6**	**154**	**1059**	**$C_{10}H_{18}O$**	**32.6**	**6.24**
10	Butanoic acid 3 methylbut-2-enyl aster	13.3	156	1068	$C_9H_{16}O_2$		6.46
11	γ- terpinene	28.9	136	1047	$C_{10}H_{16}$		6.41
12	(β- linalool)	77.1	154	1081	$C_{10}H_{18}O$	3.40	7.35
13	L- trans- pinocarveol	39	152	1143	$C_{10}H_{16}O$		8.11
14	Terpineol	25.5	154	1172	$C_{10}H_{18}O$		8.64
15	4- Terpineol	47.8	154	1161	$C_{10}H_{18}O$	5.02	8.80
16	Nerol	30.3	154	1215	$C_{10}H_{18}O$	1.73	10.15
17	1-butanol 2,3, dimethyl	11.7	102	823.9	$C_6H_{14}O$	1.89	10.37
18	2,3- Pinanediol	28.7	170	1276	$C_{10}H_{18}O_2$	1.76	11.39
19	Propanoic acid 2-methyl 2 phenylethyl ester	29.8	192	1375	$C_{12}H_{16}O_2$		12.5
20	Geraniol acetate	29.7	196	1360	$C_{12}H_{20}O_2$	1.30	12.24
21	Bicyclo(1,2,0) undec-4-ene 4,11,11-trimethyl-8-Methylene	30.4	204	1396	$C_{15}H_{24}$		12.97
22	1H cycloprop(e)azulene decahydro-1,1,7-trimethyl-4-methylene	5.83	204	1386	$C_{15}H_{24}$		13.29
23	7-isopropyl-7-methyl-nona, 3,5 diene-2,8,dione	19.8	208	1453	$C_{13}H_{20}O_2$	1.20	14.52

24	2-dodecan-1-yl succinic anhydride	19.1	266	1966	$C_{16}H_{26}O_3$	1.06	14.87
25	Vindillorol	29.6	222	1594	$C_{15}H_{26}O$		15.73
26	4a,7,7,10a-tetramethyl dodecahydrobenzol chroman-3-ol	44.7	266	1942	$C_{17}H_{30}O_2$	2.51	16.13
27	(-)spathulenol	27.9	220	1577	$C_{15}H_{24}O$		16.17
28	α- Copaen- 11-ol	12.6	220	1535	$C_{15}H_{24}O$		16.55
29	Isoaromamadendrene epoxide	21.2	220	1590	$C_{15}H_{24}O$	.208	16.84

Table-6- Larvicidal and adulticidal activity of α- pinene against *Aedes aegypti* (from pending manuscript)

α- pinene	Larvicidal	24	165.95	Y=-0.77+2.60X	2.198	3.056	100.88
		48	152.56	Y=-0.68+2.60X	2.156	3.007	63.064
		72	141.01	Y=-0.80+2.70X	2.265	3.169	73.669
	Adulticidal	24	716.55	Y=-1.17+2.15x	1.407	2.901	13.959
		48	207.78	Y=-0.4+2.33x	1.768	2.849	22.483
		72	73.30	Y=-1.76+3.62x	2.419	4.548	92.426
